# Circular RNA hsa_circ_0005909 modulates osteosarcoma progression via the miR-936/HMGB1 axis

**DOI:** 10.1186/s12935-020-01399-1

**Published:** 2020-07-13

**Authors:** Shuai Ding, Guangquan Zhang, Yanzheng Gao, Shulian Chen, Chen Cao

**Affiliations:** Department of Spine and Spinal Cord, Henan Provincial People’s Hospital, People’s Hospital of Zhengzhou University, People’s Hospital of Henan University, No. 7, Weiwu Road, Jinshui District, Zhengzhou, 450003 Henan China

**Keywords:** OS, circ_0005909, miR-936, HMGB1, OS cell lines

## Abstract

**Background:**

Osteosarcoma (OS) is the most common bone malignant tumor in children, youth, and adolescents. Circular RNA hsa_circ_0005909 (circ_0005909) is involved in the progression of OS. Nevertheless, there are few reports on the role and mechanism of circ_0005909 in OS.

**Methods:**

Quantitative real-time polymerase chain reaction (qRT-PCR) was executed to examine the expression of circ_0005909, miR-936, and High Mobility Group Box 1 (HMGB1) mRNA in OS tissues and cells. Cell viability, colony formation, migration, and invasion were evaluated by Cell Counting Kit-8 (CCK-8), cell colony formation, or transwell assays. Cell epithelial-mesenchymal transition (EMT) and HMGB1 protein levels were assessed through western blot analysis. The role of circ_0005909 on tumor growth in vivo was verified by xenograft assay. The relationship between circ_0005909 or HMGB1 and miR-936 was confirmed with the dual-luciferase reporter or RNA pull-down assays.

**Results:**

Circ_0005909 level was upregulated in OS tissues and cells. OS patients with high circ_0005909 expression had a lower survival rate. Circ_0005909 inhibition reduced tumor growth in vivo and constrained cell viability, colony formation, migration, invasion, and EMT of OS cells in vitro. Furthermore, circ_0005909 served as a sponge for miR-936 and the repressive impacts of circ_0005909 silencing on malignant behaviors of OS cells were abolished by miR-936 inhibitors. Also, HMGB1 acted as a target for miR-936 and was modulated by circ_0005909 via miR-936. Additionally, HMGB1 overexpression restored the inhibitory influence on the malignant behaviors of OS cells mediated by circ_0005909 inhibition.

**Conclusions:**

Circ_0005909 inhibition impeded the progression of OS via downregulating HMGB1 via sponging miR-936.

## Highlights

1. The expression of circ_0005909 was elevated in OS tissues and cells.
2. Inhibition of circ_0005909 decreased the malignant behaviors of OS cells.
3. Circ_0005909 acted as a sponge for miR-936 in OS cells.
4. HMGB1 served as a target for miR-936 in OS cells.
5. Circ_0005909 regulated HMGB1 expression through sponging miR-936.

## Background

Osteosarcoma (OS) is a primary bone tumor that originates from mesenchymal cells and is common in children, youth, and adolescents [1, 2]. The malignant disease is characterized by rapid progress, early metastasis, and high mortality [3]. At present, the combination of surgical resection and systemic multi-drug chemotherapy is the main method for OS treatment and the long-term overall survival rate for patients with OS is approximately 65–70% [4]. However, the overall 5-year survival rate of patients with metastasis and recurrence of OS was about 30% and 15%, respectively [5]. In consequence, exploring the molecular mechanism of OS advancement is indispensable for improving the prognosis of patients with OS.

Circular RNAs (circRNAs), a new type of endogenous non-coding RNAs, have a closed circular structure formed by covalent attachment [6]. Recently, circRNAs have been shown to interact with RNA-binding proteins and act as splicing and transcription regulators and microRNA (miRNA) sponges [7]. Also, circRNAs were associated with the advancement of tumors [8, 9]. Circular RNA hsa_circ_0005909 (circ_0005909) is derived from the reverse splicing of XPR1 mRNA and has a length of 371 nucleotides. Circ_0005909 was revealed to promote the expression of CADM1 in OS cells via acting as a sponge for miRNAs [10]. At present, the role and mechanisms of circ_0005909 in OS are rarely reported.

MiRNAs are another type of non-coding RNAs that can regulate the expression of targeted genes at the post-transcriptional level [11]. They are implicated in almost every aspects of cancer biologies, such as angiogenesis, invasion and metastasis, apoptosis, and proliferation [12]. MiR-936 was connected with the progression of epithelial ovarian cancer [13], glioma [14], and non-small cell lung cancer [15]. However, the role of miR-936 in OS has not been fully elucidated.

High Mobility Group Box 1 (HMGB1) has a dual function, it can act as a DNA chaperone inside the cells, and it works with chemokines, cytokines, and growth factors outside the cells [16]. It was reported that HMGB1 played an important role in many diseases, including cancers [17]. The genetic variation HMGB1 gene might serve as a predictor for breast cancer advancement and metastasis [18]. HMGB1 was disclosed to be connected with the progression of a variety of cancers [19–22]. Moreover, HMGB1 was involved in the sensitivity of OS to drugs and the apoptosis and proliferation of OS cells [23]. However, it is unclear whether HMGB1 can be regulated by circ_0005909 and miR-936 in OS.

Hence, we verified the role of circ_0005909 in OS. Furthermore, we also investigated the mechanism of the circ_0005909/miR-936/HMGB1 axis in OS.

## Materials and methods

### OS specimens

54 patients with OS underwent surgical treatment at Henan Provincial People’s Hospital, and their tumors tissues and neighboring non-tumor tissues were used in this study. The inclusion criteria were as follows: preoperatively confirmed as osteosarcoma by puncture or biopsy; no immunotherapy, chemotherapy, radiotherapy, or chemotherapy before surgery; patients agreed to participate in this study and clinical data were complete. The exclusion criteria were as follows: suffering from other malignant tumors; suffering from other types of serious diseases; patients received treatment for other clinical diseases within 3 months before admission; pregnant or breastfeeding. All patients informed consents before undergoing surgical resection. The association between circ_0005909 expression and clinicopathological parameters of OS patients were presented in Table 1. This research was ratified by the Ethics Committee of Henan Provincial People’s Hospital.

**Table 1:** Correlation between circ_0005909 expression and clinicopathological parameters of patients with OS

Characteristics	Number	circ_0005909 expression		*P*
		High	Low	
		27	27	
Age (years)				0.586
< 18	28	15	13	
≥ 18	26	12	14	
Gender				0.783
Male	31	16	15	
Female	23	11	12	
Tumor size (cm)				0.301
≤ 5	33	16	17	
> 5	21	11	10	
Clinical staging				0.029*
I–II	28	10	18	
III	26	17	9	
Distant metastasis				0.014*
Present	25	17	8	
Absent	29	10	19	

*P < 0.05, statistically significant

### Cell culture and transfection

Normal osteoblast hFOB1.19 and OS cell lines (HOS, U2OS, and SAOS-2) were purchased from the American Type Culture Collection (Manassas, VA, USA). All cells were kept in an incubator with 5% CO_2_ at 37 °C and cultured in Dulbecco’s modified Eagle’s medium (DMEM, Life Technologies, Grand Island, NY, USA) complemented with fetal bovine serum (FBS, 10%, Sigma, Louis, Missouri, MO, USA), streptomycin (100 μg/mL, Sigma), and penicillin (100 U/mL, Sigma).

Short hairpin RNA targeting circ_0005909 (sh-circ#1 and sh-circ#2) and negative control (sh-NC) were bought from RiboBio (Guangzhou, China). MiRNA mimics and inhibitors targeting miR-936 (miR-936 and anti-miR-936) and their negative controls (miR-NC and anti-NC) were procured from RiboBio. The sequence of HMGB1 was cloned into the pcDNA3.1 vector (vector) (Invitrogen, Carlsbad, CA, USA) to construct the overexpression vectors for HMGB1. When the cells were cultured to approximately 80% confluence, OS cells (HOS and U2OS) were transiently transfected with the assigned oligonucleotides or vectors using Lipofectamine 3000 reagent (Invitrogen) and cultured for 48 h.

### RNA preparation, RNase R digestion, and quantitative real-time polymerase chain reaction (qRT-PCR)

The nuclear and cytoplasmic RNA of OS cells were isolated with the PARIS KIT50 RXNS (Life Technologies). Total RNA was extracted through the TRIzol reagent (Invitrogen). For RNase R digestion, total RNA of OS cells was treated with RNase R (3 U/μg, Epicentre Technologies, Madison, WI, USA) at 37 °C for 15 min. Total RNA (5 μg) was reversely transcribed into complementary DNA using the PrimeScript RT reagent Kit (Takara, Dalian, China) or miRNA First-Strand Synthesis Kit (Takara). QRT-PCR was conducted through the SYBR Premix Ex Taq (Takara). The expression of circ_0005909, XPR1, miR-936, and HMGB1 was figured by the 2^−ΔΔCt^ method. The primers used in the research were displayed as below: Glyceraldehyde-3-phosphate dehydrogenase (GAPDH): (Forward: 5′-GACTCCACTCACGGCAAATTCA-3′ and Reverse: 5′-TCGCTCCTGGAAGATGGTGAT-3′); circ_0005909: (Forward: 5′-GTATCCACTTGCCCTTTA-3′ and Reverse: 5′-TTACTCCAGCCTGTCTC-3′); XPR1: (Forward: 5′-TCCACCTACGGAGGACAATC-3′ and Reverse: 5′-GGAGAAGTGCAGGCAAGAAC-3′); HMGB1 (Forward: 5′-GGGATGGCAAAGTTTTTCCCTTTA-3′ and Reverse: 5′-CACTAACCCTGCTGTTCGCT-3′); U6 small nuclear RNA (snRNA) (Forward: 5′-GCTCGCTTCGGCAGCACA-3′ and Reverse: 5′-GAGGTATTCGCACCAGAGGA-3′) and miR-936: (Forward: 5′-AACGAGACGACGACAGAC-3′ and Reverse: 5′-ACAGTAGAGGGAGGAATCGCAG-3′). GAPDH or U6 snRNA was served as an internal control.

### Cell Counting Kit-8 (CCK-8) assay

The assigned vectors or oligonucleotides were transfected into OS cells for 48 h. After washing with phosphate buffer solution (PBS), the cells were incubated with DMEM mixed the CCK-8 reagent (10 μL, Dojindo, Tokyo, Japan) for 2 h. The absorbance at 450 nm was measured through the Microplate Absorbance Reader (Bio-Rad Labs., Richmond, CA, USA).

### Cell colony formation assay

The transfected HOS and U2OS cells (1.5 × 10^2^ cells/well) were seeded in 6-well plates and maintained at 37 °C in an incubator with 5% CO_2_ for 2 weeks, and the medium was replaced every 4 days. After fixation with formaldehyde (10%, Sigma) for 20 min, cell colonies in each well were stained with crystal violet (0.1%, KeyGen, Jiangsu, China) for 5 min. The colonies (> 50 cells) were counted and photographed under a light microscope (Olympus, Tokyo, Japan).

### Transwell assay

After transfection for 48 h, the HOS and U2OS cells were collected. In the invasion assay, the transfected HOS and U2OS cells (1 × 10^5^) were seeded on the upper chamber of the transwell chamber (8 μm, Corning Costar, Corning, NY, USA) with matrigel matrix (BD Biosciences, San Jose, CA, USA). And the lower chamber of the transwell chamber was supplemented with the culture medium with FBS (10%). The cells on the upper membrane were removed after culturing for 24 h. Subsequently, the cells on the lower membrane were fixed with paraformaldehyde (4%, Beyotime, Shanghai, China) and stained with crystal violet (0.1%, KeyGen). The same procedures were used for the migration assay, but the upper chamber of the transwell chamber was not covered with the matrigel matrix. The migrated and invaded cells were figured with a light microscope (Olympus).

### Western blot analysis

Western blot analysis was executed as previously described [24]. OS tissues and cells were lysed in lysis buffer (Beyotime). After the subjection of the sodium dodecyl sulfate polyacrylamide gel electrophoresis (SDS-PAGE), the total protein was transferred onto the polyvinylidene difluoride (PVDF) membranes (Invitrogen). The membranes were probed with anti-N-cadherin (N-cad) (ab18203, 1:1000, Abcam, Cambridge, MA, USA), anti-E-cadherin (E-cad) (ab40772, 1:10,000, Abcam), anti-HMGB1 (ab79823, 1:500, Abcam), and anti-GAPDH (ab128915, 1:5000, Abcam), and then incubated with goat anti-rabbit (ab97051, 1:10,000, Abcam) immunoglobulin G (IgG). GAPDH was regarded as a loading control. Protein bands were visualized by the ImmunoStar LD (Wako Pure Chemical, Osaka, Japan).

### Xenograft assay

The animal experiment was ratified by the Ethics Committee of Henan Provincial People’s Hospital. 8 BALB/c nude mice (athymic, 4–5 weeks old) were purchased from Charles River Laboratories (Beijing, China) and were fed under specific-pathogen-free conductions. HOS cells (1 × 10^6^) with sh-NC or stable lentivirus-mediated sh-circ_0005909 (sh-circ#1, RiboBio) were subcutaneously injected into the dorsal side of the nude mice (4 mice/group). The tumor volume was measured once a week using the equation: Volume = (length × width^2^)/2. The mice were euthanized on day 35 after injection for subsequent analysis.

### Dual-luciferase reporter assay

The binding sites of miR-936 in circ_0005909 were predicted by the Circinteractome database. The sequences of circ_0005909 and mutant circ_0005909 (within the binding sites to miR-936) was inserted into the pGL3-control vector (Promega, Madison, WI, USA) for the construction of the wild type (WT) and mutant (MUT) luciferase reporter vectors for hsa_circ_0000517, respectively. The binding sites of HMGB1 in miR-936 were predicted by the Targetscan database. The same method was employed to construct the luciferase reporter vectors for HMGB1. HOS and U2OS cells were cotransfected luciferase reporter vectors and miR-NC or miR-936. The dual-luciferase reporter assay kit (Promega) was employed to assess the luciferase intensities of the luciferase reporter vectors in OS cells.

### RNA pull-down assay

In brief, the Biotinylated (Bio)-miR-NC and Bio-miR-936 from Sigma were transfected into OS cells, respectively. The lysates of OS were incubated with Dynabeads M-280 Streptavidin (Invitrogen) at 4 °C for 3 h to pull down the Bio-coupled RNA complexes. Following this, the beads were washed through the lysis buffer (Beyotime), and the complex was purified by TRIzol (Invitrogen). The enrichment of circ_0005909 in the complex was examined through qRT-PCR.

### Statistical analysis

The experiments of the research in vitro were repeated at the last 3 times. GraphPad Prism 7.0 (GraphPad, La Jolla, CA, USA) and SPSS 18.0 software (SPSS, Chicago, IL, USA) were utilized for statistical analysis. The  association between circ_0005909 expression and clinicopathological parameters of patients with OS was analyzed with the Chi square test. The survival curve was assessed via the Kaplan–Meier method and the log-rank test. The differences between the OS tissues and neighboring non-tumor tissues were analyzed by using paired Student’s *t* test. The differences between the two groups were determined by unpaired Student’s *t* test, and the differences among more groups were analyzed with one-way analysis of variance (ANOVA) followed by Tukey post hoc test. The correlation was evaluated via Pearson’s correlation analysis. Differences were deemed significant if *P* < 0.05. Data were exhibited as mean ± standard deviation.

## Results

### Expression and characteristic of circ_0005909 in OS

**Fig. 1:**
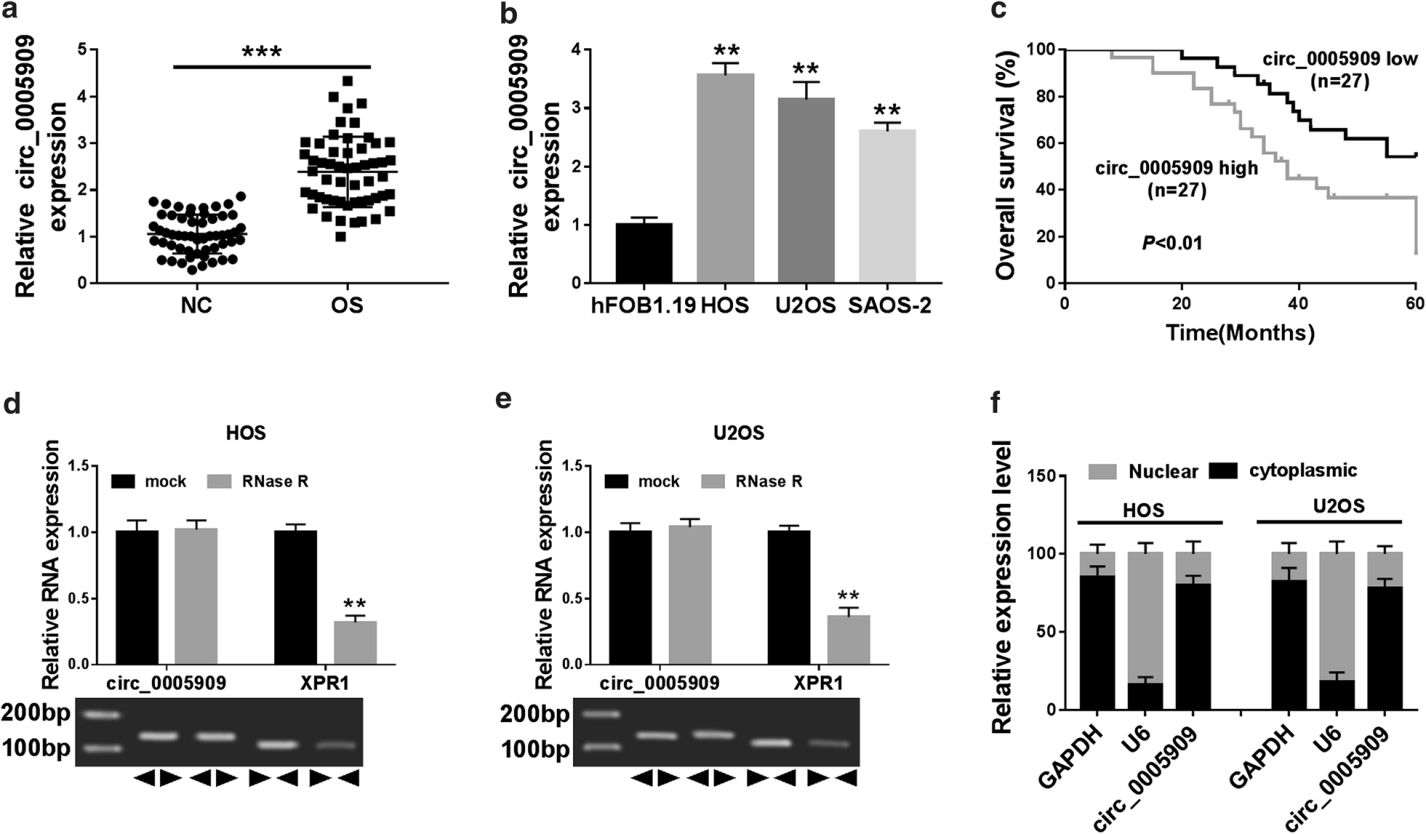
Circ_0005909 expression was enhanced in OS tissues and cells. **a**, **b** Expression of circ_0005909 in OS tissues (**a**) and cells (**b**) was analyzed through qRT-PCR. **c** The overall survival of OS patients with a high circ_0005909 expression and low circ_0005909 expression was determined via Kaplan–Meier’s curves. **d**, **e** The abundance of circ_0005909 and XPR1 in HOS and U2OS cells treated with RNase R was analyzed by qRT-PCR. **f** The abundance of circ_0005909 in the cytoplasm or nucleus of HOS and U2OS cells was assessed with qRT-PCR. **P < 0.01 and ***P < 0.001

At first, we examined the expression pattern of circ_0005909 in 54 paired OS tissues and neighboring non-tumor tissues through qRT-PCR. We observed that circ_0005909 expression was overtly increased in OS tissues with respect to that in the neighboring non-tumor tissues (Fig. 1a). Consistently, a distinct augmentation of circ_0005909 was observed in OS cell lines (HOS, U2OS, and SAOS-2) compared to the hFOB1.19 cells (Fig. 1b). Moreover, circ_0005909 expression was associated with clinical staging and distant metastasis (Table 1). Furthermore, patients with OS were divided into two groups based on the median value: high circ_0005909 expression group and low circ_0005909 expression group, and the overall survival of OS patients with the high expression of circ_0005909 were lower than the OS patients with low circ_0005909 expression (Fig. 1c). Also, compared to the liner gene XPR1, circ_0005909 was resistant to RNase R (Fig. 1d, e). Besides, we detected the relative expression of circ_0005909 in the cytoplasm and nucleus of HOS and U2OS cells. QRT-PCR displayed that circ_0005909 was primary located in the cytoplasm of HOS and U2OS cells (Fig. 1f). These findings revealed that circ_0005909 was abundant and stable in OS tissues and cells, and high circ_0005909 expression might be associated with OS advancement.

### Circ_0005909 silencing constrained viability, colony formation, migration, invasion, and epithelial-mesenchymal transition (EMT) of OS cells in vitro and repressed OS growth in vivo

**Fig. 2:**
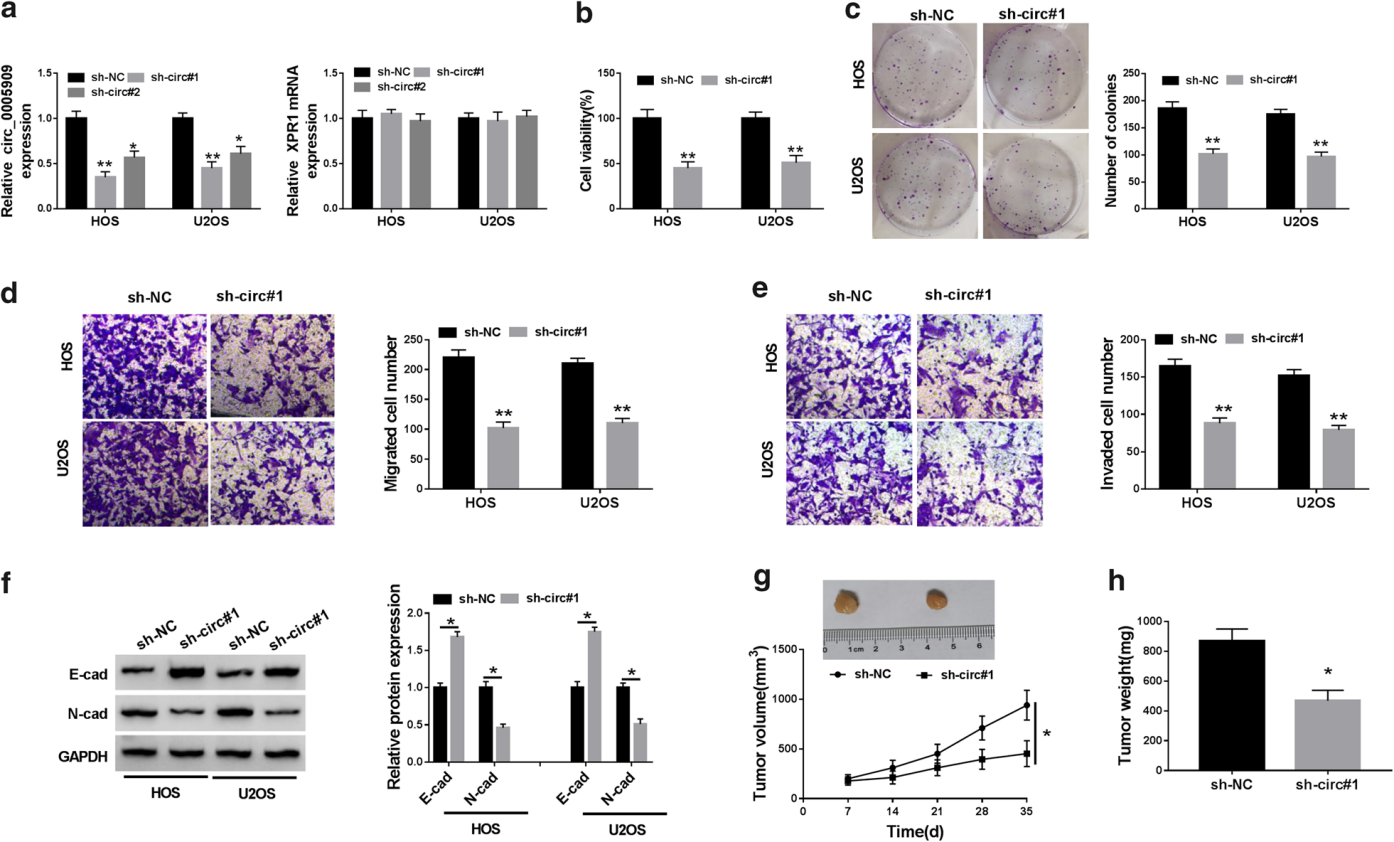
Impacts of circ_0005909 suppression on OS cell growth in vitro and in vivo. **a** QRT-PCR was executed to assess circ_0005909 and XPR1 mRNA expression in HOS and U2OS cells transfected with sh-circ#1, sh-circ#2, or sh-NC. **b**–**f** Effects of circ_0005909 downregulation on cell viability (**b**), colony formation (**c**), migration (**d**), invasion (**e**), and EMT (**f**) of HOS and U2OS cells were determined through CCK-8 assay, cell formation assay, transwell assay, or western blot analysis. **g** Tumor volume of the mice in the sh-circ#1 and sh-NC groups was measured once a week from day 7. **h** Tumor weight of the mice in the sh-circ#1 and sh-NC groups were assessed on day 35 after injection. *P < 0.05 and **P < 0.01

Considering that circ_0005909 was upregulated in OS tissues and cells, we further surveyed the role of circ_0005909 in OS through loss-function-experiments. In contrast to the control sh-NC, circ_0005909 expression was drastically reduced in HOS and U2OS cells transfected with sh-circ#1 and sh-circ#2, while the expression of XPR1 mRNA did not change (Fig. 2a). Also, the expression of circ_0005909 was lower in the sh-circ#1 group with respect to the sh-circ#2 group, so we employed the sh-circ#1 to explore the impacts of circ_0005909 silencing on viability, colony formation, migration, and invasion of OS cells. CCK-8 assay revealed that decreased circ_0005909 expression  impeded cell viability in HOS and U2OS cells (Fig. 2b). Cell colony formation assay exhibited that the colony formation ability of HOS and U2OS cells was inhibited by circ_0005909 downregulation (Fig. 2c). Transwell assay displayed that circ_0005909 reduction evidently curbed cell migration and invasion capacities in HOS and U2OS cells (Fig. 2d, e). Moreover, the EMT associated proteins, E-cad and N-cad, were detected via western blot analysis in circ_0005909-silenced HOS and U2OS cells. The results exhibited that E-cad protein expression was upregulated and N-cad protein expression was downregulated in circ_0005909-silenced HOS and U2OS cells (Fig. 2f). Subsequently, we explored the role of circ_0005909 in vivo through xenograft models. The results presented that tumor volume and weight of mice in the sh-circ#1 group were remarkably curbed compared to the control group (Fig. 2g, h). Collectively, these results indicated that circ_0005909 knockdown repressed OS cell growth in vitro and in vivo.

### Circ_0005909 acted as a sponge for miR-936 in OS cells

**Fig. 3:**
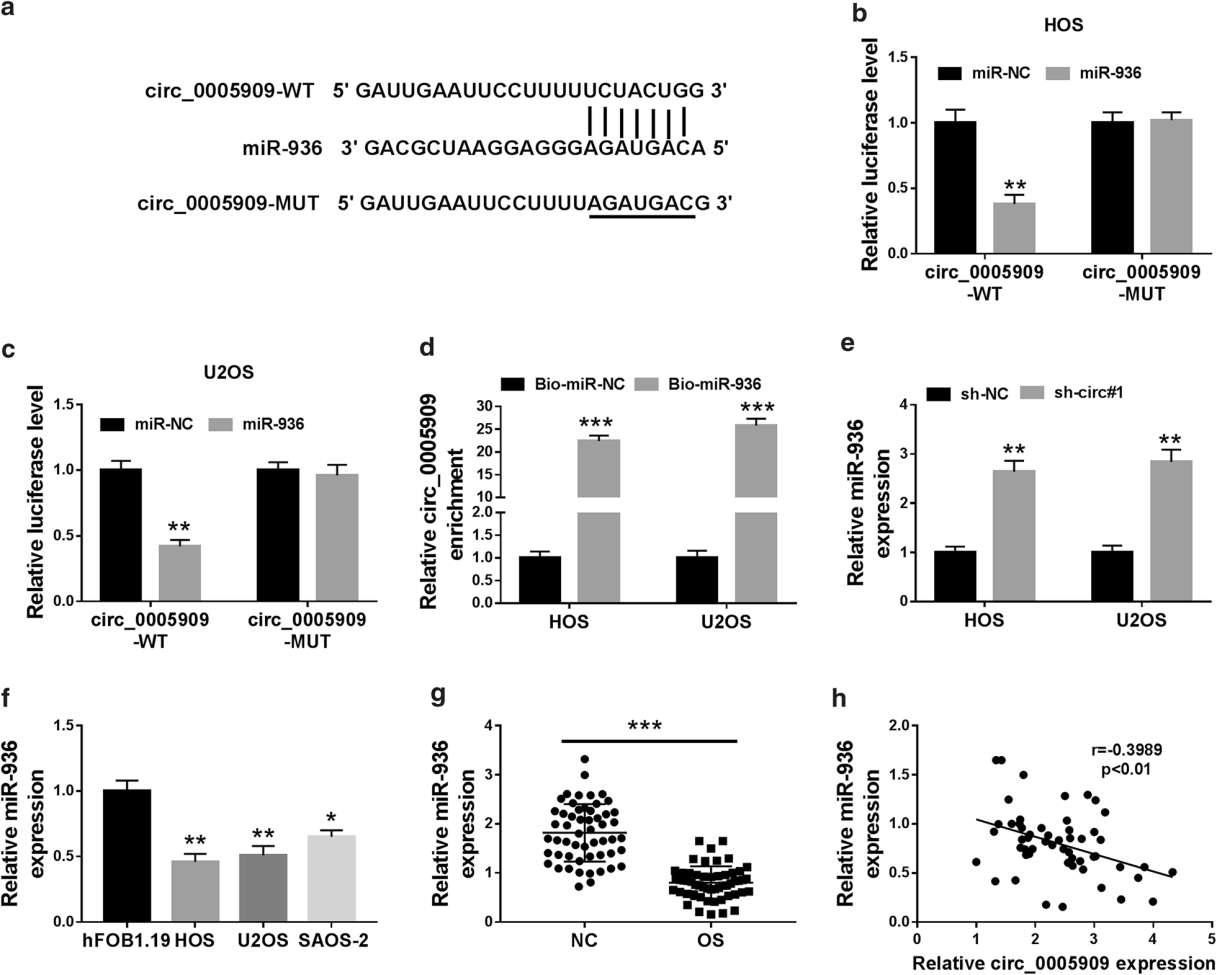
Circ_0005909 targeted miR-936 in OS cells. **a** The binding sites of miR-936 in circ_0005909 were predicted by the Circinteractome database. **b**, **c** The luciferase intensities of the circ_0005909-WT and circ_0005909-MUT reporter vectors in HOS and U2OS cells transfected with miR-936 or miR-NC were determined via dual-luciferase reporter assay. **d** The relationship between miR-936 and circ_0005909 was further verified through RNA pull-down assay. **e** Expression of miR-936 in circ_0005909-suppressed HOS and U2OS cells was examined by qRT-PCR. **f**, **g** Expression of miR-936 in OS cells (**f**) and tissues (**g**) was analyzed via qRT-PCR. **h** The correlation between miR-936 and circ_0005909 in OS tissues was assessed with Pearson’s correlation analysis. *P < 0.05, **P < 0.01, and ***P < 0.001

To investigate the molecular mechanism of circ_0005909 in OS, we predicted the target for circ_0005909 through the Circinteractome database. We discovered that 8 miRNAs (miR-338-3p, miR-411, miR-498, miR-515-5p, miR-561, miR-605, miR-766, and miR-936) had possible binding sites for circ_0005909. Silenced circ_0005909 expression could elevate the expression of miR-338-3p and miR-936 in HOS and U2OS cells, while there was no distinct difference in the other miRNAs (Additional file 1: Fig. S1). It was reported that circ_0005909 acted as a sponge for miR-338-3p in OS cells [10], so we chose miR-936 for subsequent researches. The potential binding sites between circ_0005909 and miR-936 were shown in Fig. 3a. After miR-936 mimics transfection, miR-936 expression was overtly increased in HOS and U2OS cells compared to the control group (Additional file 2: Fig S2A). Moreover, dual-luciferase reporter assay exhibited that miR-936 overexpression obviously suppressed the luciferase intensity of the circ_0005909-WT reporter vectors in HOS and U2OS cells, while the luciferase activity of the circ_0005909-MUT reporter vectors did not change (Fig. 3b, c). Also, RNA pull-down assay manifested that circ_0005909 was enriched in HOS and U2OS cells with Bio-miR-936 compared to the control group (Fig. 3d). Moreover, miR-936 expression was elevated in circ_0005909-inhibited HOS and U2OS cells (Fig. 3e). Furthermore, miR-936 expression was effectively reduced in HOS, U2OS, and SAOS-2 cells compared with the hFOB1.19 cells (Fig. 3f). The same expression trend of miR-936 was observed in OS tissues (Fig. 3g). Additionally, circ_0005909 and miR-936 had a negative correlation in OS tissues (Fig. 3h). We also explored the influence of miR-936 overexpression on viability, colony formation, migration, and invasion of OS cells. The results exhibited that miR-936 upregulation decreased cell viability, colony formation, migration, and invasion of U2OS and SAOS-2 cells (Additional file 2: Fig S2B–S2E). Together, these data indicated that circ_0005909 acted as a sponging for miR-936 in OS cells.

### MiR-936 suppression abolished circ_0005909 silencing-mediated effects on cell viability, colony formation, migration, invasion, and EMT in OS cells

**Fig. 4:**
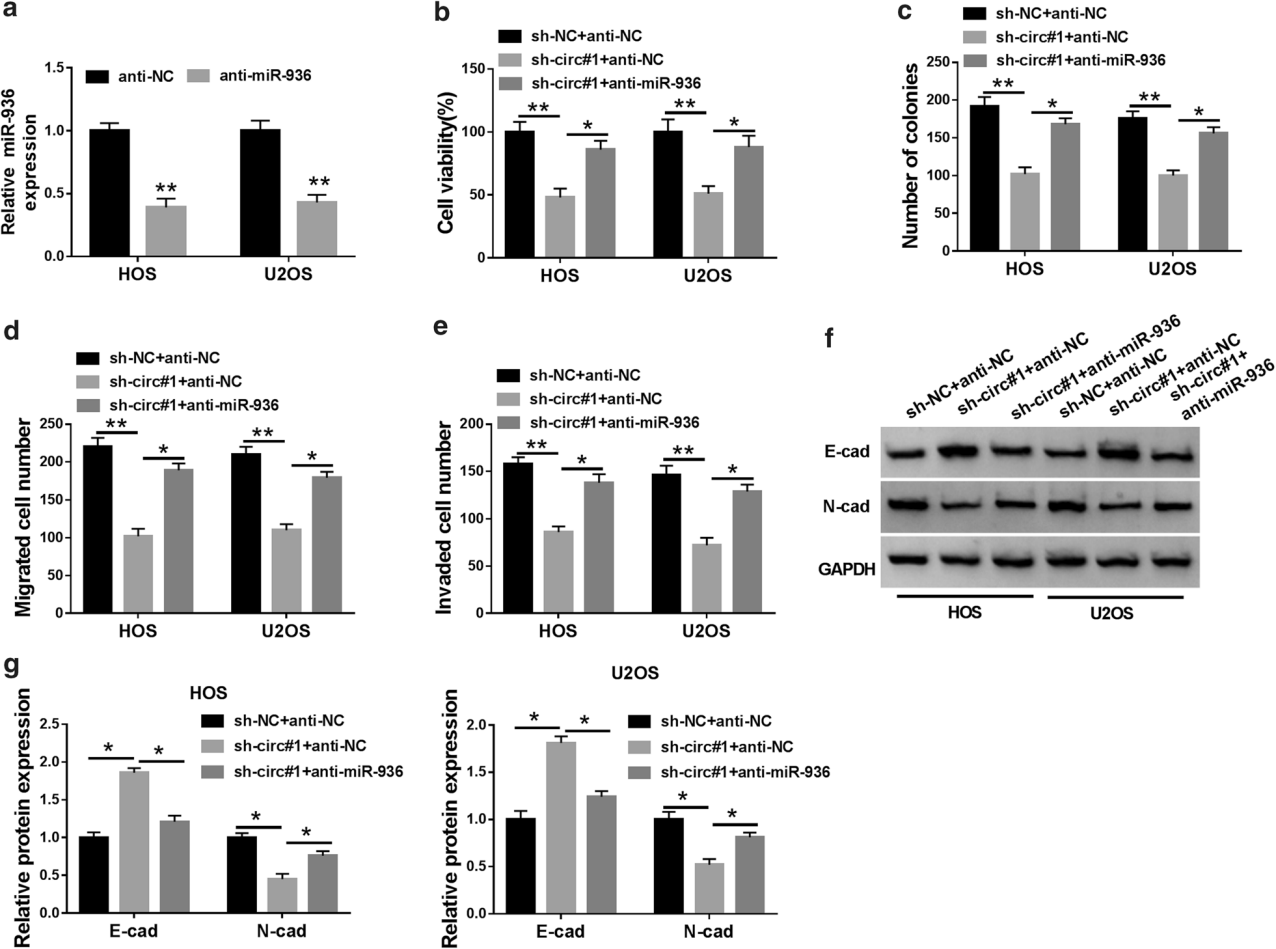
Circ_0005909 regulated cell malignant behaviors in OS cells via miR-936. **a** Expression of miR-936 in HOS and U2OS cells transfected with anti-miR-936 or anti-NC was determined with qRT-PCR. **b**–**g** Impacts of miR-936 inhibition on viability, colony formation, migration, invasion, and EMT of HOS and U2OS cells induced by circ_0005909 depletion were evaluated by CCK-8 assay (**b**), cell formation assay (**c**), transwell assay (**d**, **e**), or western blot analysis (F and G). *P < 0.05 and **P < 0.01

Given that miR-936 served as a target for circ_0005909 in OS cells, we explored whether circ_0005909 played its role via miR-936. In comparison to the anti-NC, the expression of miR-936 was curbed in HOS and U2OS cells transfected with anti-miR-936 (Fig. 4a). Moreover, the repression of viability and colony formation of HOS and U2OS cells mediated by circ_0005909 silencing was abrogated by miR-936 inhibitors (Fig. 4b, c). Also, the repressive impacts of circ_0005909 inhibition on migration and invasion of HOS and U2OS cells were overturned by miR-936 downregulation (Fig. 4d, e). Besides, silenced miR-936 expression reversed the effects of circ_0005909 inhibition on the levels of N-cad and E-cad proteins in HOS and U2OS cells (Fig. 4f, g). Taken together, these results demonstrated that circ_0005909 mediated cell viability, colony formation, migration, invasion, and EMT in OS cells via miR-936.

### HMGB1 was a target for miR-936 in OS cells

**Fig. 5:**
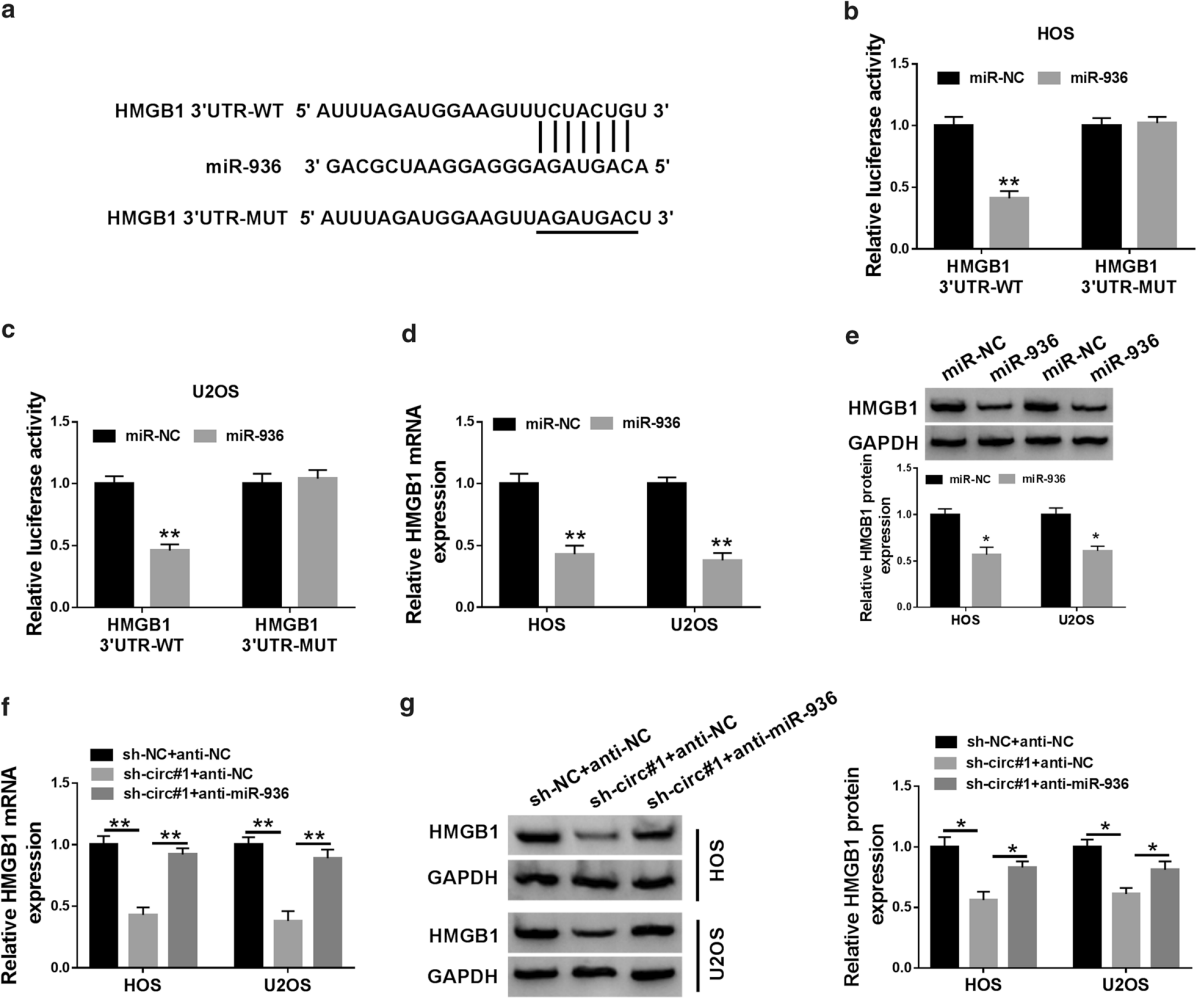
MiR-936 targeted HMGB1 in OS cells. **a** The binding sites between the 3′UTR of HMGB1 and miR-936 were predicted via Targetscan database. **b**, **c** Dual-luciferase reporter assay was carried out for the evaluation of the luciferase activities of the luciferase reporter vectors HMGB1 3′UTR-WT and HMGB1 3′UTR-MUT in HOS and U2OS cells transfected with miR-936 or miR-NC, respectively. **d**, **e** Effects of miR-936 upregulation on HMGB1 mRNA and protein expression of HOS and U2OS cells were examined via qRT-PCR or western blot analysis. **f**, **g** Influence of miR-936 suppression on HMGB1 mRNA and protein expression of HOS and U2OS cells caused by circ_0005909 downregulation was surveyed by qRT-PCR or western blot analysis. *P < 0.05 and **P < 0.01

Subsequently, we further surveyed the downstream target for miR-936 via Targetscan database. Online bioinformatics prediction (TargetScan) exhibited that 8 genes (E2F3, GSK3B, MAPK8, TWIST1, HMGB1, wmt5a, ATM, and IGF1) might be downstream targets of miR-936. Furthermore, miR-936 mimics could reduce the level of HMGB1 mRNA in HOS and U2OS cells, so that HMGB1 was selected for subsequent studies (Additional file 3: Fig. S3). As exhibited in Fig. 5a, the 3′UTR of HMGB1 had latent binding sites for miR-936. Dual-luciferase reporter assay suggested that the luciferase activity of the luciferase reporter vectors with HMGB1 3′UTR-WT was reduced in HOS and U2OS cells transfected with miR-936, while there was no overt difference in the luciferase reporter vectors containing HMGB1 3′UTR-MUT (Fig. 5b, c). Elevated miR-936 expression reduced HMGB1 mRNA and protein levels in HOS and U2OS cells (Fig. 5d, e). Also, HMGB1 mRNA and protein levels were decreased in circ_0005909-inhibited HOS and U2OS cells, while this influence was restored by miR-936 downregulation (Fig. 5f, g). Therefore, these findings disclosed that HMGB1 acted as a target for miR-936 in OS cells.

### HMGB1 elevation restored circ_0005909 downregulation-mediated influence on viability, colony formation, migration, invasion, and EMT of OS cells

**Fig. 6:**
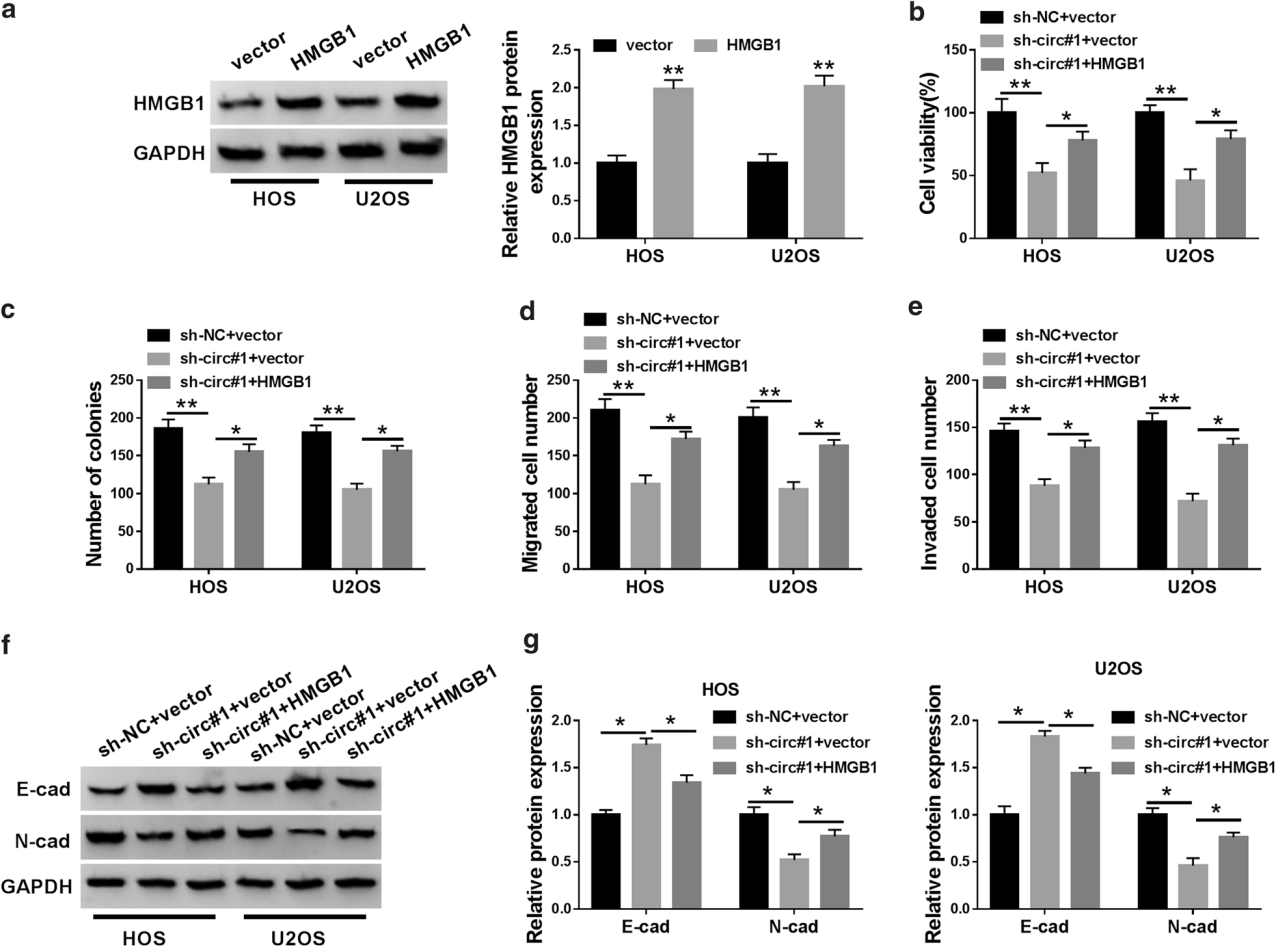
Circ_0005909 mediated cell malignant behaviors in OS cells via HMGB1. **a** Western blot analysis was conducted to assess HMGB1 protein expression in HOS and U2OS cells transfected with HMGB1 or vector. **b**–**g** Influence of HMGB1 augmentation on viability, colony formation, migration, invasion, and EMT of HOS and U2OS cells induced by circ_0005909 impediment was analyzed through CCK-8 assay (**b**), cell formation assay (**c**), transwell assay (**d**, **e**), or western blot analysis (**f**, **g**). *P < 0.05 and **P < 0.01

Knowing that circ_0005909 regulated HMGB1 expression via miR-936 in OS cells, we further surveyed whether circ_0005909 modulated cell viability, colony formation, migration, invasion, and EMT in OS cells via HMGB1. Compared to the control vectors, HMGB1 protein level was evidently increased in HOS and U2OS cells after HMGB1 transfection (Fig. 6a). Furthermore, HMGB1 elevation recovered the inhibitory impacts of circ_0005909 silencing on viability and colony formation of HOS and U2OS cells (Fig. 6b, c). Moreover, forced HMGB1 expression overturned the suppressive influence on migration and invasion of HOS and U2OS cells caused by circ_0005909 inhibition (Fig. 6d, e). Additionally, both the upregulation of E-cad and the downregulation of N-cad in circ_0005909-blocked HOS and U2OS cells were reversed by HMGB1 overexpression (Fig. 6f, g). Therefore, these results suggested that circ_0005909 modulated cell malignant behaviors in OS cells via HMGB1.

## Discussion

Accumulating researches have disclosed that circRNAs exert significant roles in different biological processes, particularly in the genesis, progression, and metastasis of tumors [25]. Also, circRNAs might be acted as promising biomarkers and targets for the diagnosis and treatment of tumors [26]. Recently, a series of researches demonstrated the importance of circRNA-mediated regulatory mechanisms on OS. For example, circRNA_100876 silencing inhibited OS progression by sponging miR-136 [27]. CircRNA circTADA2A facilitated OS metastasis and development by increasing CREB3 expression via sponging miR-203a-3p [28]. Furthermore, circRNA circ_0001785 mediated HOXB2 expression via competitively binding to miR-1200 in OS cells, which could mediate the pathogenesis of OS [29]. In this study, we revealed that circ_0005909 silencing decreased tumor growth in vivo and curbed cell viability, colony formation, migration, invasion, and EMT in OS cells in vitro. Chen et al. [10] unmasked that circ_0005909 contributed to OS progression through elevating CADM1 expression via sponging miR-338-3p. These data manifested that circ_0005909 played a promotive role in OS progression.

Given that circ_0005909 could act as a sponge for miR-338-3p in OS [10], we disocovered that circ_0005909 severed as a sponge for miR-936 in OS cells. MiR-936 was revealed to be downregulated in several cancers. Zhou et al. [15] demonstrated that miR-936 upregulation impeded E2F2 expression in non-small cell lung cancer cells, which could constrain cell cycle progression, invasion, and proliferation of non-small cell lung cancer cells. Another report indicated that miR-936 suppressed the tumor aggressiveness by inactivating the PI3K/AKT pathway via repressing FGF2 expression in epithelial ovarian cancer [13]. Furthermore, miR-936 impeded gliomas progression by negatively regulating CKS1 expression and the AKT/ERK1/2 signaling pathway [14]. Herein, miR-936 inhibition reversed circ_0005909 blocking-mediated the repressive effects on the malignant behaviors of OS cells. Therefore, we inferred that circ_0005909 modulated OS progression via sponging miR-936.

**Fig. 7:**
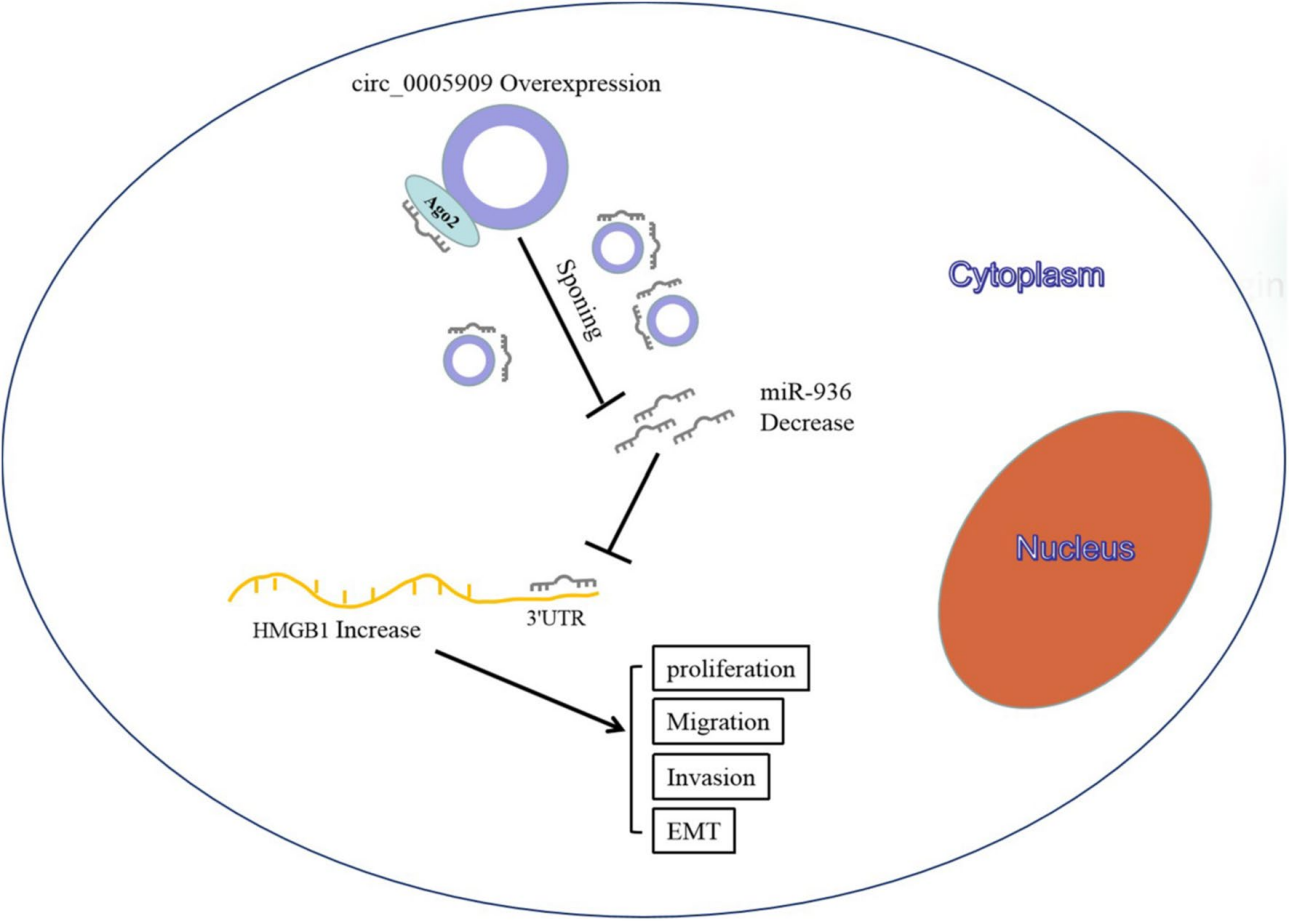
The schematic cartoon of the circ_0005909/miR-936/HMGB1 axis in OS. Circ_0005909 overexpression can sponge more endogenous miR-936 to sequester and inhibit miR-936 activity, thereby leading to the upregulation of HMGB1, which facilitating the progression of OS

Subsequently, we surveyed the downstream targets of miR-936. We found that HMGB1 acted as a target for miR-936. Previous research revealed that HMGB1 was downregulated by lncRNA MALAT1 silencing via miR-129-5p or miR-142-3p, which could induce apoptosis and curbed the growth of OS cells [30]. Moreover, Liu et al. [31] claimed that miR-505 repressed cell invasion, migration, and proliferation in OS cells via downregulating HMGB1. Also, HMGB1 silencing could reduce the resistance of OS cells to drugs [32]. We revealed that HMGB1 was modulated by circ_0005909 via miR-936. Besides, HMGB1 overexpression abolished the repressive influence of circ_0005909 silencing on the malignant behaviors of OS cells. From all the above evidence, we concluded that circ_0005909 regulated the malignant behaviors of OS cells via regulating HMGB1 expression via sponging miR-936 (Fig. 7). Additionally, in the current study, we only explored the mechanism of circ_0005909/miR-936/HMGB1 axis in OS. In the future, some signaling pathways are involved in the mechanism of circ_0005909/miR-936/HMGB1 axis and whether circ_0005909 has the ability of protein translation can be studied in depth.

## Conclusion

In all, circ_0005909 mediated the progression of OS via the miR-936/HMGB1 axis, which disclosed that circ_0005909 might be an underlying therapeutic target for OS patients.

## Supplementary information

**Additional file 1: Fig. S1.** Effect of circ_0005909 suppression on the expression of miRNAs. (A and B) QRT-PCR was executed to assess the levels of miR-338-3p, miR-411, miR-498, miR-515-5p, miR-561, miR-605, miR-766, and miR-936 in HOS and U2OS cells transfected with sh-circ_0005909#1 or sh-NC. **P < 0.01.

**Additional file 2: Fig S2.** Influence of miR-936 mimics on the malignant behaviors of OS cells. (A) After miR-936 or miR-NC transfection, the expression levels of miR-936 in HOS and U2OS cells were examined with qRT-PCR. (B-E) After miR-936 or miR-NC transfection, the viability, colony formation, migration, and invasion of HOS and U2OS cells were determined by CCK-8, cell formation, or transwell assays. **P < 0.01 and ***P < 0.001.

**Additional file 3: Fig. S3.** Impact of miR-936 mimics on the expression of its putative target genes. (A and B) QRT-PCR presented the mRNA levels of E2F3, GSK3B, MAPK8, TWIST1, HMGB1, wmt5a, ATM, and IGF1 in HOS and U2OS cells transfected with miR-936 mimics or miR-NC. **P < 0.01.

## Data Availability

The analyzed data sets generated during the present study are available from the corresponding author on reasonable request.

## Abbreviations

OS: Osteosarcoma
qRT-PCR: Quantitative real-time polymerase chain reaction
HMGB1: High Mobility Group Box 1
CCK-8: Cell Counting Kit-8
EMT: Epithelial-mesenchymal transition
WT: Wild type
