# Supplementary figures and images for: Circular RNA hsa_circ_0005909 modulates osteosarcoma progression via the miR-936/HMGB1 axis
Source: Cancer Cell Int. 2020 Jul 13;20:305. doi: 10.1186/s12935-020-01399-1 (PMC7359231; doi:10.1186/s12935-020-01399-1)

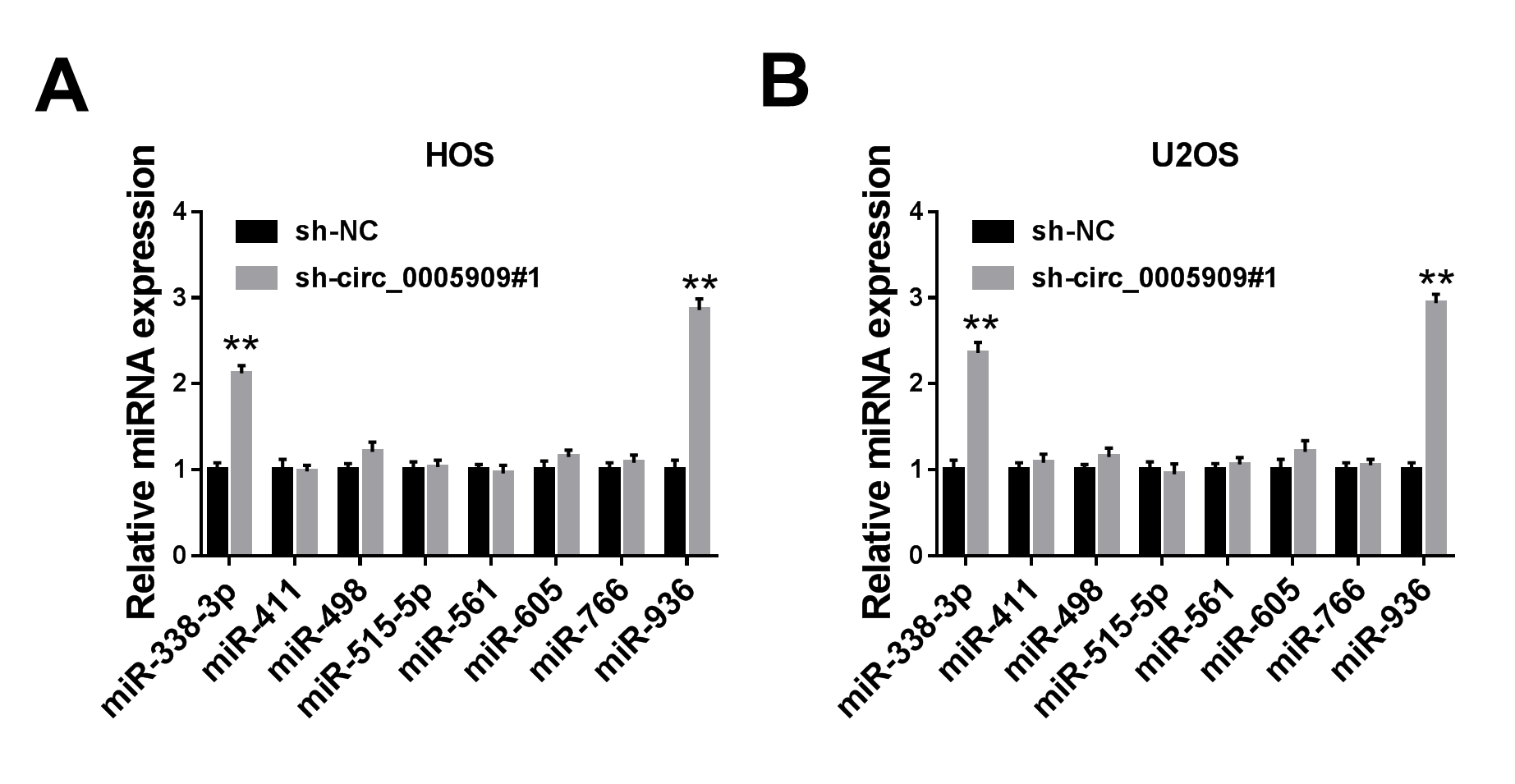

Supplement: Supplementary file 1 — Additional file 1: Fig. S1. Effect of circ_0005909 suppression on the expression of miRNAs. (A and B) QRT-PCR was executed to assess the levels of miR-338-3p, miR-411, miR-498, miR-515-5p, miR-561, miR-605, miR-766, and miR-936 in HOS and U2OS cells transfected with sh-circ_0005909#1 or sh-NC. **P < 0.01. [file 12935_2020_1399_MOESM1_ESM.tif]

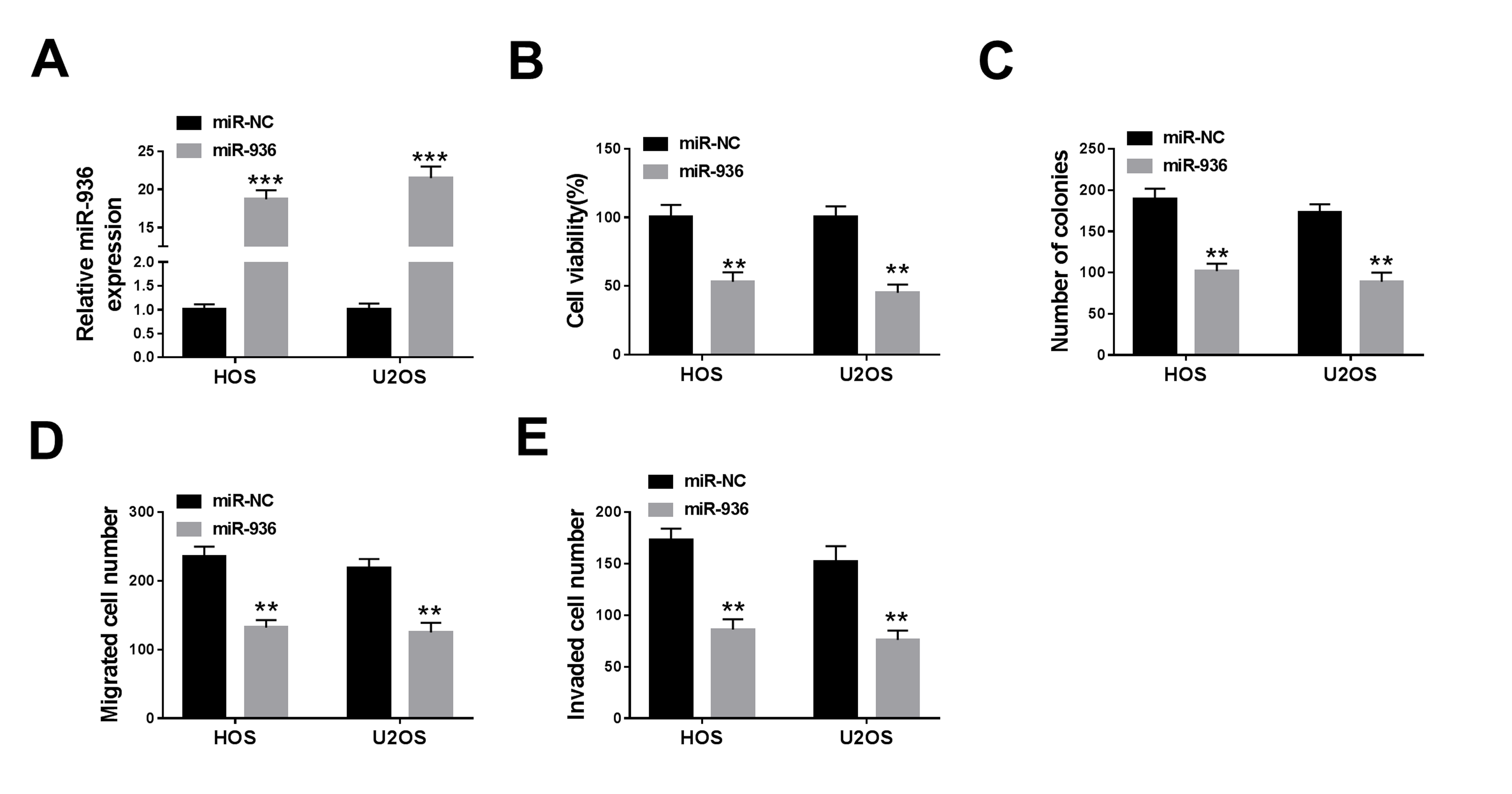

Supplement: Supplementary file 2 — Additional file 2: Fig S2. Influence of miR-936 mimics on the malignant behaviors of OS cells. (A) After miR-936 or miR-NC transfection, the expression levels of miR-936 in HOS and U2OS cells were examined with qRT-PCR. (B-E) After miR-936 or miR-NC transfection, the viability, colony formation, migration, and invasion of HOS and U2OS cells were determined by CCK-8, cell formation, or transwell assays. **P < 0.01 and ***P < 0.001. [file 12935_2020_1399_MOESM2_ESM.tif]

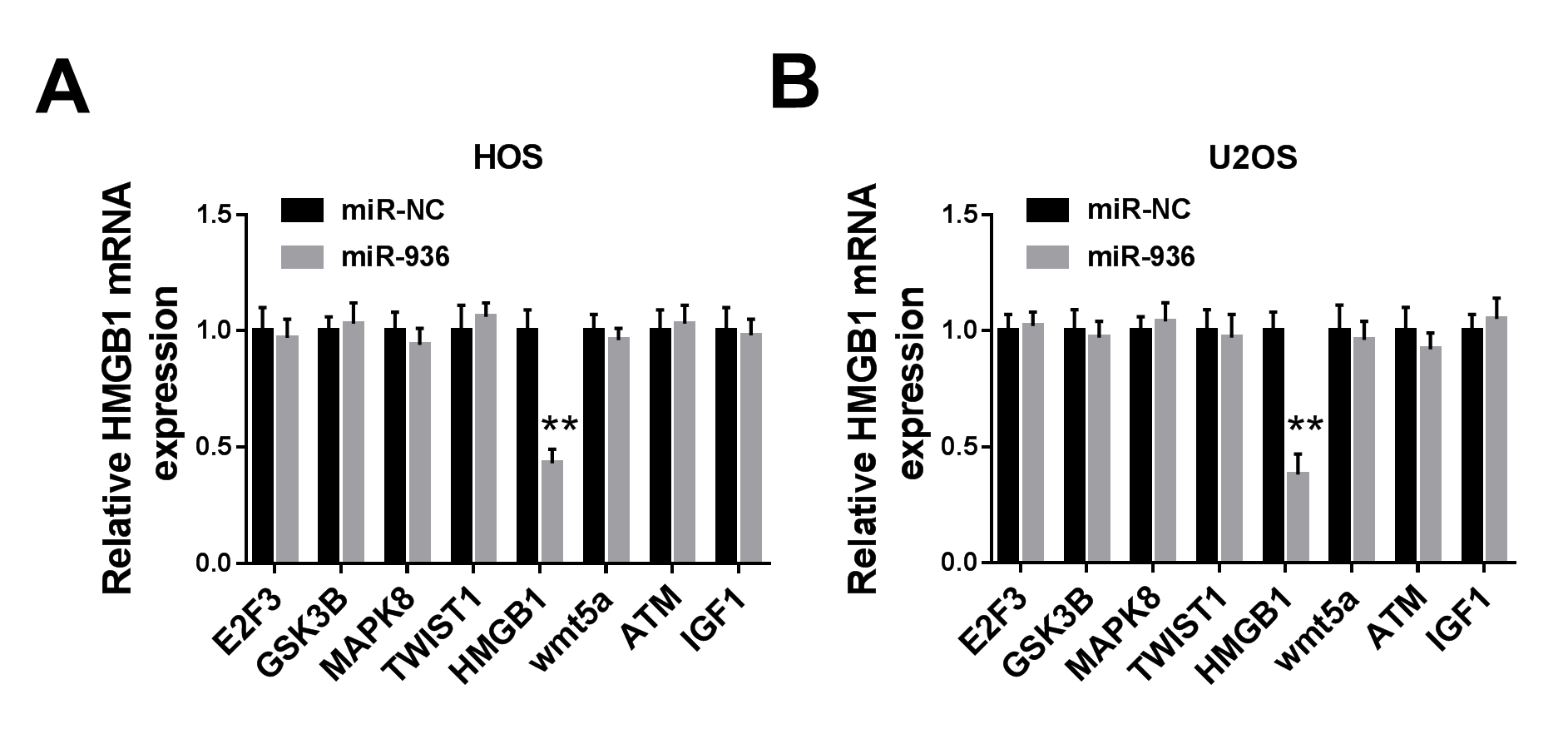

Supplement: Supplementary file 3 — Additional file 3: Fig. S3. Impact of miR-936 mimics on the expression of its putative target genes. (A and B) QRT-PCR presented the mRNA levels of E2F3, GSK3B, MAPK8, TWIST1, HMGB1, wmt5a, ATM, and IGF1 in HOS and U2OS cells transfected with miR-936 mimics or miR-NC. **P < 0.01. [file 12935_2020_1399_MOESM3_ESM.tif]
